# Polypill in heart failure: a pathway to simplified treatment and improved adherence and outcomes

**DOI:** 10.1007/s10741-025-10559-2

**Published:** 2025-09-06

**Authors:** Kalliopi Keramida, Gianluigi Savarese, Gerasimos Filippatos, Salim Yusuf

**Affiliations:** 1 Cardiology Department, General Anti-Cancer, Oncological Hospital, Agios Savvas, Athens, Greece; 2 Department of Clinical Science and Education at Södersjukhuset, Karolinska Institutet, Stockholm, Sweden; 3 Department of Cardiology, Attikon University Hospital, Medical School—National and Kapodistrian University of Athens, Rimini 1, 124 62, Athens, Greece; 4 Department of Medicine, Population Health Research Institute, McMaster University and Hamilton Health Sciences, Hamilton, Canada

**Keywords:** Heart failure, Polypill, Fixed-dose combination therapy, Adherence, Polypharmacy

## Abstract

Heart failure (HF) remains a global health challenge that imposes significant clinical and economic burden. Treatment adherence to guideline-directed medical therapy (GDMT) remains a major challenge in the management of HF, despite the availability of guideline-directed medical therapy (GDMT). Polypharmacy and regimen complexity contribute to poor adherence, particularly among older adults and in resource-limited settings. The polypill strategy, involving fixed-dose combinations of essential HF medications, has emerged as a potential solution to simplify treatment regimens, enhance adherence, and improve clinical outcomes. This review explores the potential of polypill therapy as a pragmatic strategy to simplify HF treatment and improve adherence. Drawing on its successful application in other cardiovascular diseases, we propose two implementation approaches for HF: early low-dose initiation for newly diagnosed patients or switching to a pre-specified dose polypill for stable, optimized patients. This review discusses formulations tailored to different HF phenotypes and highlights ongoing clinical trials assessing the efficacy and safety of the polypill in the HF setting. While the polypill approach offers promising benefits, i.e., improved adherence, affordability, and streamlined care, critical considerations regarding the selection of optimal drug components, identification and elimination of potential drug-drug interactions, the definition of appropriate flexible dose combinations, and patient-specific factors are crucial. Future research, particularly real-world clinical trials, is essential to comprehensively evaluate the efficacy, safety, and feasibility of polypill therapy in diverse HF patient populations, ensuring its responsible integration into clinical practice across diverse healthcare settings to mitigate the persistent burden of HF.

## Introduction

Heart failure (HF) is a clinical syndrome affecting more than 64 million people worldwide [1]. Despite the availability of guideline-directed medical treatments (GDMT) that can alter the natural course of the disease [2, 3], HF continues to impose a significant social and economic burden. This is evident in terms of years lived with disability, impact on quality of life (QoL), hospitalizations, morbidity, and mortality rates, as well as the associated healthcare costs [4–8]. The substantial burden of HF can be reduced through improved treatment adherence and implementation of GDMT [9, 10]. A low proportion of HF patients receive the full range of drug classes recommended by the guidelines and reach the target doses [11–15]. Several factors contribute to the limited implementation of HF GDMT, including polypharmacy, inadequate or no follow-up, advancing age, and multiple comorbidities [9, 16].

Polypharmacy is commonly defined as the daily use of five or more medications, while hyper-polypharmacy refers to the use of 10 or more medications [17]. Trials exploring polypharmacy and hyper-polypharmacy predominantly focus on the elderly due to the comorbidities that come with aging [18, 19]. However, polypharmacy is becoming increasingly prevalent among middle-aged individuals as well [20–22]. The term “appropriate” or “right” polypharmacy is used to acknowledge the necessary use of multiple drug therapies to manage specific or multiple medical conditions in a patient [23, 24], in contrast to “inappropriate” polypharmacy, which refers to the prescription of more medications than clinically indicated [25].

While HF alone typically requires four core medications as part of GDMT, the coexistence of other conditions—such as diabetes, atrial fibrillation, chronic kidney disease, and hypertension—and underlying pathologies such as coronary artery disease, substantially increases medication burden and contributes to the clinical and adherence challenges associated with polypharmacy [26]. Remarkably, 90% of HF patients have three or more comorbid conditions, with 50% having five or more [27]. A recent systematic review revealed a high prevalence of polypharmacy in HF, ranging from 17.2 to 99% [28]. The incidence of polypharmacy and hyper-polypharmacy is increasingly notable among elderly HF patients [26], with 41% receiving more than 10 medications at hospital discharge between 2003 and 2006, rising to 68% between 2011 and 2014 [29].

The evidence-based therapy for HF with reduced ejection fraction (HFrEF) consists of four pillars: angiotensin-converting-enzyme inhibitors (ACEi), angiotensin receptor blockers (ARBs), or angiotensin receptor-neprilysin inhibitors (ARNIs); beta-blockers (BBs); mineralocorticoid receptor antagonists (MRAs); sodium-glucose co-transporter-2 inhibitors (SGLT2i) [2, 3]. Additional medications may be used based on the clinical scenario, including diuretics for decongestion and symptom improvement, ivabradine for heart rate control, antiplatelets or anticoagulants, hydralazine, isosorbide dinitrate, antiarrhythmics, and others, thereby increasing the number of daily medications used. Furthermore, novel drugs such as vericiguat, which have shown a significant impact on outcomes in HF patients, may be added to the aforementioned medications [30, 31].

Polypharmacy in HF carries several risks, including an increased likelihood of rehospitalizations for HF [32], a higher risk of all-cause mortality [33], a negative impact on patients’ experience, occurrences of adverse drug events, and significant financial burden [34]. Polypharmacy significantly impairs the QoL and is associated with non-adherence, which in turn contributes to increased hospitalizations and worse clinical outcomes in HF [35–40]. Adherence is defined as “the extent to which the patient follows medical instructions given for prescribed treatment” [41]. Non-adherence to prescribed medication has been observed in up to 45% of patients [36]. Adherence is particularly poor in low- and middle-income countries, but differences within Europe have also been observed, highlighting global regional and socio-economic disparities [36, 42].

Polypharmacy also affects patients’ persistence with HF medications, due to several reasons, including the complexity of the therapeutic regimen with frequent adjustments, adverse reactions, lack of understanding, and financial burden. The implications of discontinuation of HF therapy are related to worse QoL, increased hospitalizations, and mortality.

A promising strategy to address polypharmacy and improve treatment adherence is the use of a polypill. A polypill is a fixed combination of multiple drugs in predefined, usually low doses, contained within a single pill or capsule. The low doses typically refer to half or less of a standard dose, particularly for antihypertensive medications. The concept of combining multiple drugs into a single pill for the secondary prevention of cardiovascular disease (CVD) was initially introduced by the World Health Organization (WHO) in 2001 [43], followed by Yusuf S. a year later [44]. However, the term “polypill” therapy was coined in 2003 by Wald and Law [45], suggesting that it could potentially reduce CV events by 80% in both primary and secondary prevention by prescribing it to individuals aged 55 years and older. The first polypill proposed by Yusuf S. included aspirin, a beta-blocker (BB), a statin, and an ACEi, while Wald and Law proposed a six-element polypill containing three antihypertensives, aspirin, a statin, and folic acid.

Polypill, also known as fixed-dose combination therapy, has been utilized to address the challenges of polypharmacy and improve adherence to multidrug therapy in various contexts such as initiation, step-up, or substitution indications [46]. Polypills have been extensively tested for the treatment of different disease entities, including HIV [47], malaria, and tuberculosis [48], as well as cerebrovascular disease [49]. However, the majority of studies evaluating polypills have focused on CVD, demonstrating positive results in both primary and secondary prevention [50–58]. In addition to assessing efficacy, several clinical trials have also examined the safety of polypills [51, 59, 60], as well as their role in improving adherence, leading to better outcomes [51, 53, 57, 60–62]. Notably, the benefit in adherence has been found to be more significant in patients with lower baseline adherence. In the UMPIRE (use of multidrug pill in reducing cardiovascular events) trial, the improvement in adherence with the polypill was significantly greater among participants who had poor adherence at baseline, leading to larger reductions in blood pressure and LDL cholesterol [51]. Similarly, the FOCUS study (Fixed-Dose Combination Drug for Secondary Cardiovascular Prevention) demonstrated that patients with lower adherence at enrollment experienced the most substantial gains in medication-taking behavior after switching to a polypill-based strategy [50].

**Fig. 1:**
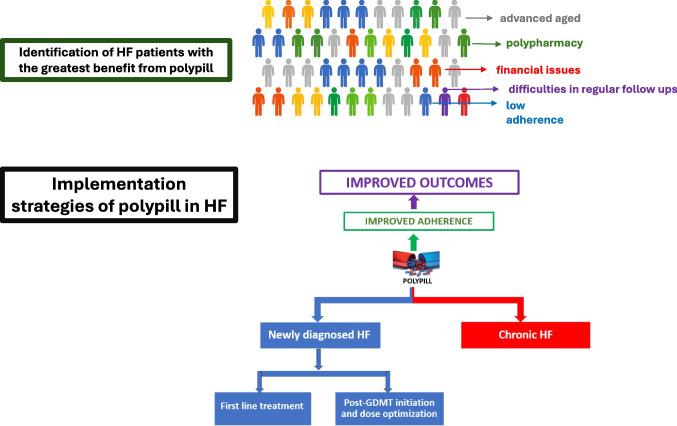
Target population and implementation strategy for polypill in heart failure

The utilization of a polypill in HF, proposed also by a recent position paper by the Heart Failure Association (HFA) of the European Society of Cardiology for addressing polypharmacy in HF [63], could offer a simplified treatment approach, replacing multidrug therapy with sequential initiation and titration. This pragmatic implementation strategy aims to address undertreated patients, increase adherence and persistence, and improve outcomes. Two main approaches could be considered for implementing a polypill in HF. Firstly, a low-dose polypill could be prescribed at the time of newly diagnosed HF with the potential for subsequent up-titration, using polypills with varying doses of the individual components [64]. Alternatively, in stable HF patients after optimizing the dose of each medication according to HF guidelines, a switch to a polypill containing the pre-specified doses could be implemented (Fig. 1). Regardless of the scenario, meticulous monitoring of renal function, blood pressure, and electrolytes is crucial, especially during the initial weeks of polypill use, to promptly detect and manage any potential adverse events (e.g., hypotension, hyperkalemia). This approach aims to balance treatment efficacy with patients' adherence, recognizing the diverse needs and challenges within the HF population.

**Fig. 2:**
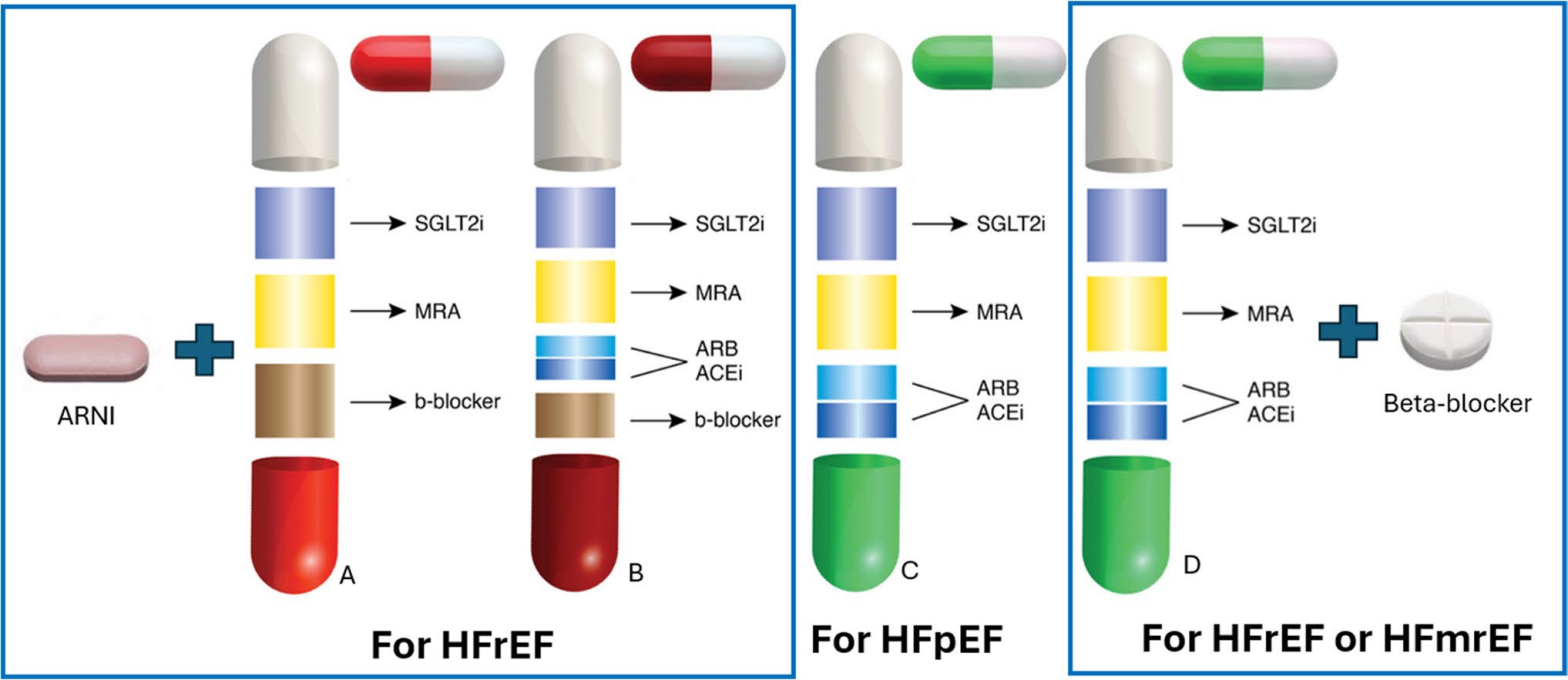
Proposed compositions of polypill for heart failure with reduced, mildly reduced and/or preserved ejection fraction

The composition of the polypill can be similar across the spectrum of left ventricular ejection fraction, or it may vary depending on whether it is intended for HFrEF or for HF with preserved ejection fraction (HFpEF). In the first case, a common polypill containing an ACEi/ARB, a SGLT2i, and a MRA can be used alone for patients with HFpEF in accordance with the most recent guidelines (Fig. 2C) [3, 65] or in combination with a beta-blocker in patients with HFrEF and HFmrEF (Fig. 2D). In the second case, for HFrEF, the polypill should ideally include a beta-blocker, an MRA, and an SGLT2i, in different combinations of doses, as recommended by GDMT [2, 3] (Fig. 2A). This polypill can be combined with an ARNi. A second formulation of polypill, for low- and lower-middle-income countries or populations generally, could also include an ACE inhibitor or an ARB, since the cost of ARNIs—which could be prescribed on top of the polypill—is very high (Fig. 2B). Notably, ongoing trials are currently evaluating polypills in HFrEF, including a trial with a three-agent polypill (metoprolol succinate, empagliflozin, and spironolactone) (NCT04633005) and another with a four-agent polypill (beta-blocker, SGLT2 inhibitor, spironolactone, and ACE/ARB/ARNI) (NCT06029712) [66] (Table 1).

**Table 1:** Ongoing trials with polypill in heart failure

Clinicaltrials.gov ID	Brief title	Type of polypill	Primary endpoint
NCT04633005	**Polypill strategy for heart failure with reduced ejection fraction**	Spironolactone 12.5 mg, empagliflozin 10 mg, and 25 mg, 50 mg, 100 mg, or 150 mg of metoprolol succinate	To determine whether the use of a polypill for HFrEF leads to higher left ventricular ejection fraction (LVEF) compared with usual care in a low-income population
NCT06029712	**Heart failure polypill at a safety net hospital**	Beta-blocker, SGLT2 inhibitor, spironolactone, and ACE/ARB/ARNI	The primary outcome will be adherence to overall and individual components of GDMT at 4, 8, 12, and 16 weeks, as measured by pill count

In both ESC 2021 and AHA/ACC/HFSA 2022 HF guidelines, patients with HFmrEF are considered eligible for the same pharmacological therapies recommended for HFrEF [2, 3]. While these therapies carry a class IIb recommendation in HFmrEF due to the lower level of evidence, their inclusion reflects a pragmatic approach, especially considering the phenotypic overlap between HFmrEF and HFrEF and the use of these therapies in everyday clinical practice as reflected in major registries. Similarly, MRAs are included in HFpEF formulations based on the IIb recommendation in patients with elevated natriuretic peptides and structural heart disease [3]. The selective non-steroidal MRA finerenone reduced the risk of cardiovascular outcomes in patients with HF and ejection fraction > 40% in the FINEARTS trial [67]. The HF events have been also reduced in subgroup analyses of patients with chronic kidney disease and diabetes with and without HF in the FIDELIO-DKD [68] and FIGARO-DKD [69] trials, as well as in the pooled analysis FIDELITY [70]. The recent approval of finerenone by the regulatory authorities will probably change management and polypill strategy for this population in the near future.

The identification of appropriate HF patients for polypill therapy is crucial. Data from the ASIAN-HF registry support that the majority of HFrEF patients (70.3%) are eligible for a polypill [71]. The ideal candidate for polypill therapy in HF is a patient who has reached clinical stability and tolerates core GDMT agents, but who may face barriers to ongoing optimization due to factors such as low adherence, socioeconomic constraints, or limited follow-up infrastructure (Fig. 1). While basic monitoring is essential during early use, the polypill may be particularly valuable in consolidating therapy for patients who have already undergone titration or are unable to adhere to complex multi-drug regimens (Fig. 1). In such cases, it can serve as a pragmatic solution to preserve long-term adherence and mitigate care fragmentation. Older patients, who constitute the majority (around 80%) of the HF population [72], often present with a significant number of cardiac and non-cardiac comorbidities, frailty, lower tolerance to higher medication doses, and a higher risk of adverse events. This age group is particularly susceptible to polypharmacy and the resulting non-compliance, making them suitable candidates for polypill therapy.

However, the successful implementation of polypill therapy in HF will require addressing several system-level and structural factors. Firstly, it is crucial to select the most effective agent within a specific class of drugs to be included in the polypill. Apart from efficacy, careful attention should be given to potential drug-drug interactions, both between the agents included in the polypill and with commonly prescribed medications in geriatric HF patients, such as selective serotonin reuptake inhibitors for depression, proton pump inhibitors, antibiotics, or anticancer medications. Once the selection of agents is made, defining the various combinations and doses becomes essential.

Clinical trials are necessary to assess the bioequivalence, pharmacokinetics, pharmacodynamics, and possibly efficacy and safety of the polypill, particularly in patients with HFpEF and HFmrEF, for whom evidence remains limited. While early studies are ongoing, robust data supporting widespread use are still lacking. Ultimately, it is essential to advocate for pharmaceutical companies to develop and distribute affordable, low-cost HF polypills, ensuring broader accessibility for diverse populations.

Moreover, the development and availability of HF polypills face considerable logistical and regulatory challenges. These include complex approval pathways, manufacturing scalability, and ensuring stable fixed-dose combinations of drugs with differing pharmacologic profiles. In low- and middle-income countries—where the heart failure burden is high but healthcare resources and follow-up infrastructure may be limited—the polypill could offer a pragmatic solution. However, implementation may be hindered by fragmented drug ownership, as guideline-recommended agents are often patented by different pharmaceutical companies. Overcoming such barriers may require coordinated efforts through cross-industry collaborations, public–private partnerships, and policy-level incentives. In addition, digital health tools and virtual care platforms could enhance adherence monitoring, enable remote titration strategies, and support safer implementation in older or underserved populations. While this review primarily focuses on the clinical rationale and patient-level considerations, we acknowledge that these broader factors are critical for the real-world adoption and equitable integration of polypill strategies into diverse healthcare systems. Although specific cost-effectiveness data for polypills in heart failure are currently lacking, recent studies in cardiovascular prevention suggest that polypill strategies can offer high economic value in underserved populations, supporting the potential for scalable implementation. For example, a recent economic evaluation based on the Southern Community Cohort Study (SCCS) Polypill Trial found that a cardiovascular polypill priced at $463 per year yielded an incremental cost-effectiveness ratio (ICER) of $8560 per quality-adjusted life-year (QALY) gained compared with usual care, qualifying as high-value care in 99% of modeled scenarios [73].

Possible clinical limitations of polypill therapy in HF include:

-Increased risk of adverse events: Polypill therapy may carry a higher risk of adverse events, such as hyperkalemia and hypotension. These could inadvertently result in premature or inappropriate de-escalation of GDMT, with potential consequences for outcomes. Therefore, the design of polypill formulations should prioritize low initial doses, particularly for vulnerable patients, and their implementation must be accompanied by careful monitoring and individualized follow-up.
-Obligatory discontinuation in case of adverse events: If a specific adverse event can be attributed to one of the agents in the polypill, discontinuation of the entire polypill may be necessary. However, this can result in non-adherence when patients are switched back to multiple individual pills.
-Limited flexibility in combinations and titration options: Polypills offer fewer options for flexible combinations and titration compared to individual medications. While this simplicity may help counteract clinical inertia and decision fatigue, it may not provide the same level of personalized treatment optimization.
-Non-compliance and its impact on outcomes: Non-compliance with the polypill regimen may have a more significant impact on outcomes compared to cases where a patient forgets or stops taking a single medication. Ensuring patient education and monitoring adherence is crucial for the success of polypill therapy.

To proactively mitigate these limitations and ensure the safe and effective use of polypill therapy, strategies aligned with comprehensive medication management in HF, as proposed by the HFA of the ESC, are essential [63]. These include periodic medication reviews to reassess appropriateness and optimize treatment, pharmacist-led medication reconciliation, and interdisciplinary collaboration to support safe deprescribing and prevent the accumulation of redundant or harmful therapies. This is particularly relevant in older adults with HF, where medications such as aspirin for primary prevention or allopurinol in normouricemic patients may often be safely deprescribed following individual benefit–risk assessment [74]. Furthermore, emerging tools such as digital adherence tools and virtual care platforms show promise in offering timely feedback on adverse effects, assisting with treatment monitoring, and improving medication-taking behaviors, particularly in older or frail populations [63].

This review, in contrast to the recent HFA position paper on polypharmacy in heart failure [63], aims to advance the discussion by specifically exploring the role of polypill therapy as a structured approach to treatment simplification. While the HFA paper appropriately highlights medication reconciliation, deprescribing, and therapeutic prioritization, it does not address polypill-based strategies or their potential for standardized implementation. Our review proposes tailored polypill configurations based on HF subtypes and patient characteristics, emphasizing both clinical applicability and socioeconomic feasibility as complementary tools to current polypharmacy guidance. The use of a polypill for HF has the potential to simplify treatment strategies, improve adherence, enhance affordability, and address global and socioeconomic disparities in the optimal implementation of evidence-based HF treatments. However, the efficacy, efficiency, safety, tolerability, and feasibility of polypill therapy in HF require thorough exploration through dedicated real-life clinical trials.

## Data Availability

No datasets were generated or analyzed during the current study.
